# Skin Burns from Monochloroacetic Acid Leak in a Chemical Plant: a Case Report

**DOI:** 10.2478/aiht-2020-71-3401

**Published:** 2020-06-29

**Authors:** Yiming Tao, Tingting Liu, Xiangdong Jian

**Affiliations:** 1 Qilu Hospital of Shandong University, Department of Poisoning and Occupational Diseases, Jinan, China

**Keywords:** chemical burn, continuous renal replacement therapy, occupational accident, poisoning, kemijske opekline, kontinuirana bubrežna nadomjesna terapija, profesionalno otrovanje

## Abstract

The patient, a 45-year-old male chemical factory worker, was burned by monochloroacetic acid discharged from a ruptured pipe. The patient was merely flushed with water and did not leave the workplace immediately. As a result, he suffered local burn symptoms, which gradually worsened. Two and a half hours after the accident, he developed symptoms of systemic poisoning, such as lethargy and dyspnoea. After a thorough debridement of the wound surface and subsequent skin grafting combined with early glucocorticoid therapy and haemofiltration, a satisfactory result was achieved, and the patient eventually recovered. With the widespread use of monochloroacetic acid in China, incidents of poisoning with this chemical are becoming increasingly common, with more than 100 cases reported in the past ten years in China alone.

## Abstract (hr)

Radnik u kemijskoj tvornici u dobi od 45 godina zadobio je opekline izazvane monokloroctenom kiselinom koja je iscurila iz napukle cijevi. Ranu je samo isprao vodom i nije odmah napustio radno mjesto. Zbog toga je imao lokalizirane simptome opekline koji su se s vremenom pogoršavali. Dva i pol sata nakon nesreće pojavili su se simptomi sistemskog otrovanja poput letargije i zaduhe (dispneje). Cjelovito uklanjanje oštećenoga tkiva s površine i presađivanje kože u kombinaciji s ranim liječenjem glukokortikosteroidom i hemofiltracijom bilo je uspješno i bolesnik se naposljetku oporavio. S raširenom primjenom monokloroctene kiseline povećan je i broj otrovanja, koji je u posljednjih deset godina samo u Kini dosegnuo broj veći od 100 slučajeva.

Monochloroacetic acid (CAS number:79-11-8) is increasingly used as an intermediate or raw material in a large number of chemical products. Production, use, handling, and transportation of monochloroacetic acid is associated with a growing number of cases of acute poisoning caused by accidental inhalation or skin burns. Monochloroacetic acid can enter the Krebs cycle and convert into citric acid ester chloride, which can then inhibit the aconitic acid enzyme system (1). Inhalation or skin contact with monochloroacetic acid may damage the heart, lungs, liver, kidneys, and central nervous system within hours after poisoning (2, 3). Severe, life-threatening consequences of exposure to monochloroacetic acid include convulsions, coma, shock, kidney failure, and severe acidosis.

This report has two primary objectives. The first is to detail the clinical course of a severe case of monochloroacetic acid poisoning to expand current medical knowledge of this dangerous condition. The second is to promote awareness of hazards associated with the production of monochloroacetic acid, which continues to increase in China.

## Case description

A 45-year-old man (height: 174 cm, weight: 82 kg) with no relevant medical history or known substance abuse had been working in a chemical plant producing and storing monochloroacetic acid for almost 20 years. The containers and pipes used for production, use, storage, and transportation of monochloroacetic acid in the factory are made of corrosion-resistant materials. However, they had not been inspected and maintained for a long time. On 17 October 2019, at 10 am, a glass tube ruptured as 80 % monochloroacetic acid, heated to 60 °C, was being delivered under pressure. The liquid sprayed the patient’s chest, left armpit, and right wrist. Although he immediately took off the clothes, redness, swelling, and the sensation of burning appeared in the affected area of the skin. The patient rinsed the acid off with water for 15 min and dried the skin with a towel. He then reported the accident and waited for assistance at his workplace. He complained of monochloroacetic acid mist and smell in the workshop and inhaled some of the vapours of the acid. Shortly thereafter, pain appeared in the area that previously was in contact with monochloroacetic acid, and dyspnoea and irritating dry cough developed. The patient was rushed to the local hospital in an ambulance, and during the ride, the skin was washed with a 4 % solution of sodium bicarbonate. Despite this treatment, the skin continued to show redness, ulceration, and blisters, and some areas turned dark brown. He arrived at the hospital approximately 2.5 h after the accident, and emergency debridement of necrotic areas was performed immediately. Shortly after, the patient developed lethargy, dyspnoea, and hypoxemia and was transferred to the Qilu Hospital for treatment the following day (18 October 2019). Upon admission, the patient’s vital signs were as follows: blood pressure (BP) 152/104 mmHg, heart rate (HR) 90 beats/min, respiratory rate (RR) 13 breaths/min, body temperature 37 °C, and oxygen saturation 95 % (with oxygen treatment at the rate of 5 L/min). The patient was conscious, lethargic, and developed dry heaving and blurred vision. Crepitations were audible over the lung base bilaterally. Heart rhythm was normal, the abdomen was flat and soft, and no abnormality was apparent. Table 1 shows the laboratory findings on admission to the Qilu Hospital.

**Table1:** Laboratory findings upon the patient’s admission to Qilu Emergency Department (18 October 2019)

Parameter	Laboratory results	Reference range
WBC	**10.41**	3.5–9.5
CK (U/L)	**1351**	38–174
CK-MB (ng/mL)	**6.9**	0.3–4.0
Cr (μmol/L)	67	58–133
K^+^ (mmol/L)	3.90	3.5–5.0
Na^+^ (mmol/L)	140	137–145
ALT (IU/L)	**2281**	21–72
AST (IU/L)	**2065**	15–59
CTNI (ng/L)	**897.90**	<30
pH	7.37	7.35–7.45
pCO_2_ (mmHg)	**31**	35–45
pO_2_ (mmHg)	88	80–100
Blood lactate (mmol/L)	**2.4**	0.5–1.8
Urine occult blood	**positive**	negative

WBC – white blood cells; CK – creatine kinase; K^+^ – serum potassium; Na^+^ – serum sodium; Cr – serum creatinine; ALT – alanine aminotransferase; AST – aspartate aminotransferase; CK-MB – creatine phosphokinase isoenzyme; CTNI – cardiac troponin I; pCO_2_ – partial pressure of carbon dioxide; pO_2_ – partial pressure of oxygen

**Figure 1:**
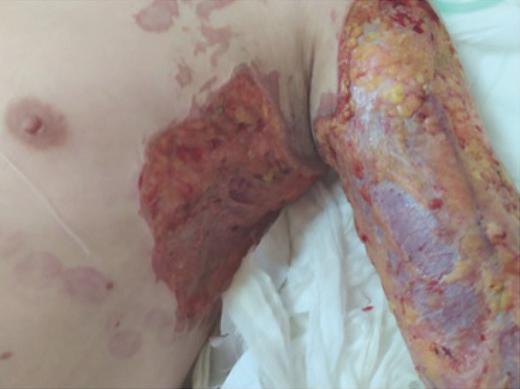
Monochloroacetic acid burn wound

**Figure 2:**
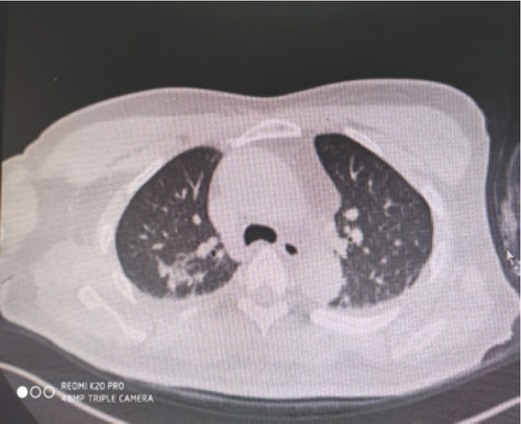
Lung CT performed on 24 October 2019, seven days after the accident, showing a few regions of inflammation in both lungs, fibrous foci, a small amount of effusion in the bilateral pleural cavity, and swelling of adjacent pulmonary tissue

After administration of anti-tetanus immunoglobulin, the wound (Figure 1) was treated again by repeated rinsing with iodophor and saline and covering with antibacterial dressing. An intravenous infusion was initiated to maintain water-electrolyte balance and provide antibiotic treatment with cefoperazone/sulbactam and moxifloxacin. The patient also received daily intravenous infusions of 200 mg methylprednisolone as pulse therapy. Considering the large area of the burn and the long time during which monochloroacetic acid was absorbed, bedside continuous veno-venous haemofiltration (CVVH) was applied every other day to rapidly remove acid from the circulation and prevent acute renal failure. Patient’s lethargy gradually improved, and the level of consciousness returned to normal on the third day of treatment. The wound dressing was changed daily, and the patient did not have severe acidosis, oliguria, or anuria. However, he developed a cough with purulent yellow sputum and his temperature rose to 38.8 °C but dropped to normal after the administration of antibiotics. Six days after the admission (on 24 October), the patient’s condition was relatively stable, and laboratory tests improved (Table 2). Wheezing, coughing, and expectoration continued, however. Figure 2 shows the head, abdomen, and chest scan (Figure 2) revealed cerebellar infarction, a few ischemic degeneration foci in the brain, a few inflammation areas in both lungs, fibrous foci, a small amount of effusion in bilateral pleural cavity, and adjacent pulmonary tissue swelling, indicating monochloroacetic acid aspiration pneumonia. The patient therefore continued to receive antibiotics and started a regular terbutaline atomisation therapy to help expel the phlegm, and the patient was encouraged to cough out the sputum.

**Table2:** Laboratory findings on 24 October and 5 November 5

Parameter	24 October	5November	Reference range
WBC	**14.99**	8.62	3.5–9.5
CK (U/L)	131	116	38–174
CK-MB (ng/mL)	3.7	**10.3**	0.3–4.0
Cr (μmol/L)	**54**	64	62–115
K^+^ (mmol/L)	4.36	3.87	3.6–5.0
Na^+^ (mmol/L)	139	142	137–145
ALT (IU/L)	**257**	**73**	9–50
AST (IU/L)	34	36	15–40
CTNI	**72.26**	**47.51**	<30
Urine occult blood	negative		negative

WBC – white blood cells; CK – creatine kinase; K^+^ – serum potassium; Na^+^ – serum sodium; Cr – serum creatinine; ALT – alanine aminotransferase; AST – aspartate aminotransferase; CK-MB – creatine phosphokinase isoenzyme; CTNI – cardiac troponin I

**Figure 3:**
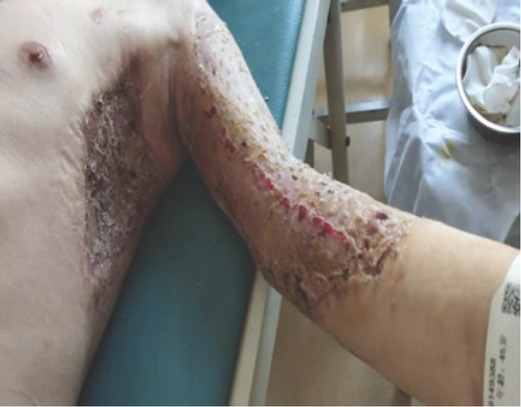
Satisfactory outcome of skin grafting

The patient remained stable until 5 November (see blood test findings in Table 2). Methylprednisolone was discontinued and replaced with oral prednisone, which was gradually reduced. Given the stable condition of the patient, the burn department was contacted to arrange skin grafting. The patient was diagnosed with a 10 % third-degree burn. On 18 November, he underwent debridement and negative pressure suction of the upper limbs and left chest wall under general anaesthesia. Granulation and stale tissue on the wound surface were removed. Skin grafting was performed under general anaesthesia by rinsing the wound with hydrogen peroxide and sterile saline, and implanting the patient’s left anterior thigh full-thickness flap into the left upper limb and the left side of the chest wall. The wound healed perfectly (Figure 3), and the patient was discharged on 4 December 2019. The results of the tests performed during a follow-up visit on 29 April 2020 were within the normal range.

## Discussion

Considering that acute poisoning with monochloroacetic acid progresses rapidly, has high mortality rate, and that its global production is about four million tonnes a year, it has raised great concern among emergency and occupational health specialists. In 2001, the European Centre for Ecotoxicology and Toxicology of Chemicals (ECETOC) published therapy guidelines (2), and in 2011, the Chinese Ministry of Health issued similar guidelines to protect the health of workers exposed to monochloroacetic acid and to standardise the diagnosis and treatment of acute monochloroacetic acid poisoning (4).

Pure inhalation poisoning through the respiratory tract is rare, and transdermal absorption is the main cause of acute poisoning (2, 5). As a strong acid, monochloroacetic acid is corrosive to the skin and produces irreversible damage to the eyes (2). When the skin is burned, the capillaries under the skin dilate, accelerating the absorption. Experiments with a daub of ^14^C-labeled monochloroacetic acid applied to rat skin demonstrated that upon skin absorption, the concentration of monochloroacetic acid in the heart, lungs, and muscles peaked after 45 minutes and two hours (6, 7). This time course is similar to that seen in humans. In the two previously reported cases (1, 8) and the present case, the clinical signs of systemic poisoning generally developed at one to three and a half hours after the exposure. The time to the appearance of symptoms was directly related to the concentration of monochloroacetic acid, duration of exposure, and skin area in direct contact with the acid (9). In the early stage of poisoning, some patients experience nausea and vomiting, while at later stages, the involvement of the central nervous system (CNS) is predominant (9, 10). Contamination of more than 5 % of the skin can cause poisoning and even death (11).

As monochloroacetic acid can block the Krebs cycle and damage essential organs by converting to citric acid ester chloride, which inhibits the aconitic acid enzyme system (1), attention should be paid not only to the treatment of burn wounds but also to the treatment of systemic poisoning. First, the contaminated clothing should be quickly removed and the skin wiped with clean gauze, rinsed with a 4 % solution of sodium bicarbonate, and then with saline (14, 15). Thorough debridement of the wound should follow, and then an early escharotomy and delayed skin graft in case of deep wounds (16, 17). These steps are critical, since early escharotomy can remove the surface of the tissue that contains monochloroacetic acid and reduce further corrosion of the tissue. The delay in skin grafting provides sufficient time to dilute and eliminate monochloroacetic acid that penetrates the tissue and to improve the survival rate of skin grafts, accelerate wound healing, and ensure better appearance and function of the limb after the wound has healed (18). As the wounds caused by monochloroacetic acid burns cause extreme expansion of muscle blood vessels, which, in turn, accelerates the absorption of the chemical and aggravates poisoning, early haemodialysis should be used as part of the treatment (2,8). Depending on the urgency of the patient’s condition, treatment should also include continuous renal replacement therapy, glucocorticoid administration, correction of acidosis, and shock prevention. The focus is on the protection of the myocardium, prevention of cardiovascular damage, control of brain oedema, and preservation of the kidney function. If myoglobinuria occurs, haemodialysis should be combined with plasmapheresis. Pulmonary oedema should be actively prevented and treated by inhalation.

The results of animal experiments demonstrated that dichloroacetic acid is the most effective drug against monochloroacetic acid poisoning (2, 3), as it acts directly on the affected enzymatic system. Moreover, dichloroacetic acid can reduce or eliminate the effects of lactic acid poisoning and is well-tolerated by humans. In Sweden it has already been approved as an antidote for life-threatening monochloroacetic acid poisoning, and in the US this indication is at the clinical trial stage. However, in most countries it has not yet been approved for this indication.

The accident described in this case report has taught us several lessons: 1) chemical companies should pay more attention to the risk and prevention of pipe leaks and provide adequate ventilation at the workplace; 2) stricter operating rules and regulations should be introduced and implemented for replacement and cleaning of the pipe system; 3) personal protection equipment, such as effective gas masks and protective clothing should always be used; 4) first aid kits and emergency showers should be readily available at sites where chemical burns may occur; 5) training in regulations and occupational protection should be regularly provided to the employees; and 6) a chemical accident emergency rescue network should be established.

According to the patient’s own statement, he had received safety training for employees working with monochloroacetic acid. However, he did not pay attention to proper procedures, and some protective measures were not implemented. Although Chinese laws and regulations regarding prevention and treatment of occupational diseases have clear provisions on protection and management in the manufacturing process, in reality some small private enterprises do not follow these regulations to their full extent. At present, China’s National Health Commission has set up the Occupational Health Department to further strengthen the supervision of occupational health and has included safety training and emergency rescue capability for enterprises as key assessment indicators. It is believed that incidents such as the one reported here will be dramatically reduced in the future.
